# Prevalence of first-degree atrioventricular block and the associated risk factors: a cross-sectional study in rural Northeast China

**DOI:** 10.1186/s12872-019-1202-4

**Published:** 2019-10-07

**Authors:** Zhi Du, Liying Xing, Min Lin, Yuanmeng Tian, Li Jing, Han Yan, Boqiang Zhang, Shuang Liu, Shiwen Yu, Yingxian Sun

**Affiliations:** 1 Department of Cardiovascular Medicine, The First Hospital of China Medical University, Shenyang, 110001 Liaoning China; 2 Disease Control and Prevention Centre of Liaoning Province, Shenyang, Liaoning China; 3 Department of Cardiovascular Medicine, Benxi Central Hospital, Benxi, Liaoning China; 4 Department of Cardiovascular Ultrasound, The First Hospital of China Medical University, Shenyang, Liaoning China; 5 Department of Periodontics, School of Stomatology, China Medical University, Shenyang, Liaoning China

**Keywords:** First-degree atrioventricular block, Risk factors, Electrocardiography, Epidemiology

## Abstract

**Background:**

First-degree atrioventricular block (AVB) has traditionally been regarded as a benign condition but recent studies have challenged this conception. Prevalence of 1–2% have been reported in developed countries in Asia. However, no epidemiologic studies have established the prevalence of first-degree AVB in developing countries. The aim of the study was to investigate the prevalence of first-degree AVB in rural northeast China and identify the associated risk factors.

**Methods:**

This cross-sectional study was undertaken from September 2017 to May 2018 in rural areas of Liaoning Province. It involved 10,926 participants aged ≥40 years (85.3% of those who were eligible). First-degree AVB was confirmed by at least two independent cardiologists. Risk factors were evaluated using stepwise logistic regression.

**Results:**

The prevalence of first-degree AVB was 3.4% (95% confidence interval [CI]: 3.0–3.8%). Males had a higher prevalence than females (5.1% vs. 2.2%, *p* < 0.001). The regression model involving all participants showed that age (odds ratio [OR]: 1.32; *p* <0.001), male sex (OR: 1.72; *p* = 0.001), height (OR: 1.25; *p* = 0.008), systolic blood pressure (SBP) (OR: 1.15; *p* = 0.003), triglycerides (TG) (OR: 1.10; *p* < 0.001), high-density lipoprotein cholesterol (HDL-C) (OR: 0.73; *p* < 0.001), heart rate (OR: 0.78; *p* < 0.001), and exercising regularly (OR: 0.73; *p* = 0.030) were independent risk factors.

**Conclusions:**

First-degree AVB is highly prevalent in rural areas of northeast China. The associated independent risk factors include being male, older, and taller, higher SBP and TG, lower HDL-C and heart rate, and lack of exercise.

## Background

First-degree atrioventricular block (AVB) is defined as abnormal prolongation of the PR interval (> 0.20 s), which is frequently encountered in clinical practice [1]. Several studies suggest that first-degree AVB appears to be a benign condition, although these studies overwhelmingly focus on young and healthy males [2–4]. However, a growing body of literature has challenged the established conceptions regarding the apparently “benign” echocardiogram (ECG) finding. Schnabel et al. and Cheng et al. have reported that PR interval prolongation in the Framingham cohort is predictive of atrial fibrillation (AF) development, pacemaker implantation, and all-cause mortality [5, 6]. A recent meta-analysis of 14 observational studies involving 400,750 individuals further suggests an association between PR interval prolongation (meeting the criteria for first-degree AVB) and significant increases in AF, heart failure, and death [7]. Furthermore, genome-wide association studies show that the genetic determinants of PR interval prolongation overlap with those of many cardiovascular diseases, [8, 9] which explains the abovementioned associations from a genetic perspective. Therefore, identifying up-to-date prevalence trends and determinants is important given the potential effects of first-degree AVB and its associated health complications.

The prevalence of first-degree AVB, which is 1–2% among the general population in developed countries [4, 5, 10–12], is associated with race [13]. In Asia, the prevalence of first-degree AVB has only been reported for Japan (PR interval ≥ 0.22 s) [11] and Korea (only in patients with hypertension) [14]. No epidemiologic studies have established the prevalence of first-degree AVB in developing countries in Asia. China, the most populous developing country, has distinct geographical features, climate features, and lifestyles that differ from those in other countries and, more specifically, so does the rural northeast region of China. For example, most people in rural northeast China are physical laborers engaged in heavy manual work. The purpose of this study was to identify the prevalence of first-degree AVB and the contributing risk factors in the population of rural areas in northeast China.

## Methods

**Fig. 1:**
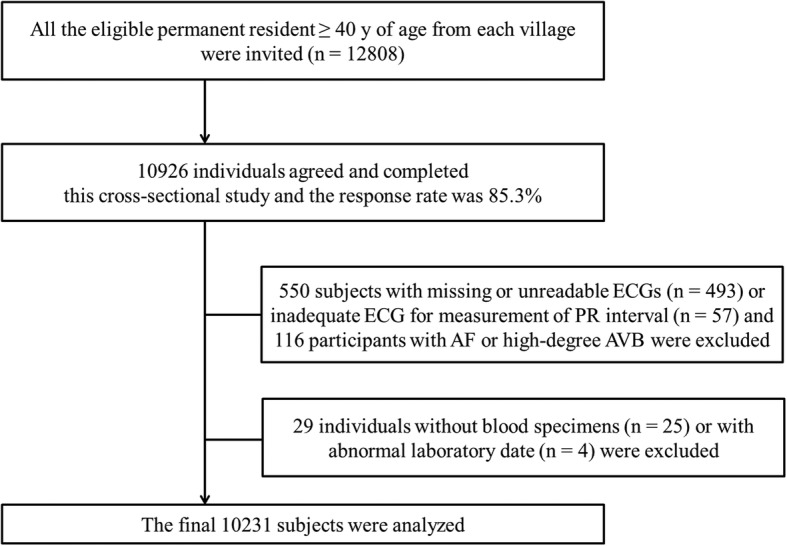
Study flow chart

This cross-sectional study was conducted in rural areas of Liaoning Province from September 2017 to May 2018. The design and enrolment process of the study has been previously described [15, 16]. Briefly, using a multistage, stratified, cluster randomized sampling strategy, four different counties were randomly selected distributed across the eastern, central and western regions of Liaoning Province, namely Liaoyang, Chaoyang, Lingyuan, and Donggang, and then nineteen rural villages in the four counties were randomly selected for inclusion in the study. All permanent residents aged at least 40 years old from the chosen villages (*n* = 12,808) were invited to participate, but subjects who were pregnant or had mental disorders were excluded, and 10,926 participants (response rate 85.3%) completed the investigation. We further excluded participants (*n* = 695) for the following reasons: missing or unreadable ECGs or inadequate ECGs for measurement of the PR interval (*n* = 550), AF or high-degree AVB (*n* = 116), lack of blood samples (*n* = 25), and abnormal laboratory data (*n* = 4). Eventually, data from a total of 10,231 (93.6%) subjects (4050 men and 6181 women) were analyzed (Fig. 1).

The study was granted approval by the Central Ethics Committee at the China National Center for Cardiovascular Disease. Written informed consent from all study participants was obtained.

The relevant data were collected by a survey team, composed of dedicated cardiologists and neurologists and specialists from the Center for Disease Control and Prevention. Two survey modes -- self-administered questionnaire and face-to-face structured interview were examined during a single clinic visit. Furthermore, the surveyors were strictly trained before starting data collection and the inventory content, operative procedure and methods were standardized, and pilot interviews with volunteers were completed.

The demographic and clinical data, including age, gender, lifestyle (smoking, drinking, and physical activity), socioeconomic status (education, occupation, and annual household income), comorbidities (hypertension, diabetes and dyslipidemia), and other medical history (cerebrovascular disease and coronary artery disease) were recorded through the self-administered questionnaire. When a patient self-reported a specific medical history, the clinicians reviewed the patient’s medical records to confirm the report. Each enrolled individual if he or she had a history of a specific disease (such as diabetes) and then asked to answer the question “Have you ever been diagnosed with [specific disease] by a medical specialist? In order to data authenticity and reliability, double-check were executed to removed unqualified answer questionnaire and irrelevant information by trained staff. Participants were asked whether they regularly consumed alcohol, their average alcohol consumption per day, and the number of days per month that they consumed alcohol. They were divided into four categories: never drank, moderate drinkers, heavy drinkers, and former drinkers. One drink was defined as containing 15 g of ethanol [17]. Moderate drinking was defined as up to 1 drink/day for women and up to 2 drinks/day for men; heavy drinking was defined as > 1 drink/day for women and > 2 drinks/day for men [18]. The definition of regular exercise was moderate exercise, which amounts to 30 min of walking at least 3 times a week [19]. Lack of exercise was defined as failing to meet the conditions above for regular exercise.

Physical data, including height, weight (the nearest 0.1 kg), and waist circumference (the nearest 0.1 cm) were obtained during the visit to the clinic. The body mass index (BMI) was defined as the ratio weight (kg) and the square of the height (m^2^). To ensure data were got according to standardized protocols, a central steering committee with a subcommittee for quality control was established.

Blood samples were collected from participants who had fasted for at least 8 h. Samples were drawn from antecubital vein into BD Vacutainer tubes containing ethylenediaminetetraacetic acid (EDTA; Becton, Dickinson and Company, Franklin Lakes, NJ, USA), and centrifuged at 3000 rpm for 5 min. The supernatant was collected and stored at − 20 °C until use. Subsequently, biochemical parameters, including fasting blood glucose (FBG), glycosylated hemoglobin (HbA1c), triglyceride (TG), total cholesterol (TC), low-density lipoprotein cholesterol (LDL-C), and high-density lipoprotein cholesterol (HDL-C), were assayed using an Abbott Diagnostics C800i auto-analyzer (Abbott Laboratories, Abbott Park, IL, USA) with commercial kits. These laboratory examinations were repeated three times at different laboratories. In order to ensure the accuracy of test results, 10% randomly selected samples in each laboratory was reexamined by National Center for Clinical Laboratory of the Ministry Health of China.

Twelve-lead ECGs (resting, 10 s) were obtained for each volunteer through a MAC 5500 System (GE Healthcare; Little Chalfont, UK). All ECGs were analyzed manually by at least two well-trained cardiologists with the assistance of magnifier and calipers. The PR interval was defined as the interval from the onset of the P wave (junction between the T–P isoelectric line and the beginning of the P wave deflection) to the end of the PR segment (junction with the QRS complex). A single lead (lead II) was used and the PR interval was determined as the mean measure from three consecutive beats or two consecutive beats at slower heart rates (< 50 beats per min). ECG-based diagnoses (including first-degree AVB and AF) were confirmed by at least two independent cardiologists. Heart rate and QRS interval data were also collected.

For each participant, blood pressure was measured using a standardized automatic electronic sphygmomanometer (J30; Omron, Kyoto, Japan) after at least 5 min of rest in a seated position, and a total of three times at 2-min intervals. In addition, participants were asked about whether corresponding medications for blood pressure, blood glucose, and blood lipids control had been taken in the last 2 weeks. If they responded “Yes”, they were asked to identify the name, dosage, and frequency of that drug if known, and those who were not entirely sure specific dose should clarify the number of tablets or pill taken. We cleaned and matched all the names of generic medicines in the China Pharmacopoeia 2015, with a 95% success rate.

On an ECG recording, First-degree AVB was defined as a PR interval > 0.2 s. Hypertension was defined as a mean systolic blood pressure (SBP) ≥140 mmHg and/or a mean diastolic blood pressure (DBP) ≥90 mmHg, and/or self-reported use of antihypertensive medication within 2 weeks [20]. Dyslipidemia was diagnosed if the individuals met one or more of the following criteria: (1) serum TC level ≥ 6.22 mmol/L; (2) serum TG level ≥ 2.27 mmol/L; (3) serum LDL-C level ≥ 4.14 mmol/L; (4) serum HDL-C level < 1.04 mmol/L; or (5) self-reported use of lipid-regulating medications over the previous 4 weeks. Elevated or decreased blood lipid status was determined according to the cut-off values mentioned above. Diabetes mellitus was diagnosed as an HbA1c ≥ 6.5% or FPG ≥ 7.0 mmol/L, and/or self-reported physician-confirmed diagnosis [21]. Cerebrovascular diseases (such as ischemic or hemorrhagic stroke) were diagnosed by a neurologist according to the World Health Organization recommendations and confirmed with computed tomography (CT) and/or magnetic resonance imaging (MRI) [22]. ECG-based left ventricular hypertrophy (ECG-LVH) diagnoses were made on the basis of ECG Rv1 + Sv5 or Rv1 + Sv6 values > 4.0 mV for males and > 3.5 mV for females. AF was diagnosed by any medical history of AF from referring physicians and/or current ECG findings.

### Statistical methods

Descriptive statistics were calculated for all study variables. Continuous variables followed a normal distribution were presented as means and standard deviations. Otherwise, continuous variables are reported as medians and upper and lower quartiles. Student’s t test and the nonparametric Mann–Whitney U test were used, as appropriate, to compare differences in continuous variables between participants with and without first-degree AVB. Categorical variables are reported as frequencies and percentages in each subgroup. Chi square tests were used to compare differences in categorical variables between participants with and without first-degree AVB. The overall and age- and gender-specific prevalences of first-degree AVB were calculated.

Non-stratified and sex-stratified univariate and stepwise multivariate logistic regression analyses were performed to determine the associations between selected demographic and clinical characteristics and first-degree AVB. Potentially significant risk factors (according to the non-stratified univariate logistic regression analyses) were added to the non-stratified and sex-stratified stepwise multivariate regression equations, including age, sex, smoking and drinking status, regular exercise status, height, weight, SBP, DBP, TG, LDL-C, HDL-C, and heart rate. In a sensitivity analysis involving the male participants, we further adjusted for cerebrovascular disease. Odds ratios (ORs) and 95% confidence intervals (CIs) are presented for the logistic regression analyses.

Statistical analyses were performed using SPSS software version 22.0 (SPSS Inc., Chicago, IL, USA); *p*-values < 0.05 were considered statistically significant.

## Results

Of the 10,231 subjects, 348 had first-degree AVB. The participants’ clinical characteristics by first-degree AVB status are shown in Table 1. Compared with the subjects without first-degree AVB, those with first-degree AVB were significantly older, taller, and heavier and had significantly higher SBP, DBP, TG, LDL-C, and QRS interval (all *ps* < 0.05). In contrast, the subjects with first-degree AVB had significantly lower HDL-C and heart rate (all *ps* < 0.05). In addition, the group with first-degree AVB had a significantly higher proportion of men, individuals with hypertension, dyslipidemia, smokers, drinkers, and individuals who exercised regularly (all *ps* < 0.05). However, there were no significant differences in other factors between subjects with and without first-degree AVB.

**Table 1:** Characteristics of the study sample

Variable	Without first-degree AVB (*N* = 9883)	With first-degree AVB (*N* = 348)	*P* value
Age, years	59.68 ± 9.98	62.76 ± 10.55	< 0.001
Male	3841 (38.9)	209 (60.1)	< 0.001
Smoking status			
Current smoker	2525 (25.5)	121 (34.8)	< 0.001
Former smoker	848 (8.6)	43 (12.4)	
Never smoked	6510 (65.9)	184 (52.9)	
Drinking status			
Never drank	6949 (70.3)	212 (60.9)	< 0.001
Moderate drinker	1777 (18.0)	94 (27.0)	
Heavy drinker	933 (9.4)	39 (11.2)	
Former drinker	224 (2.3)	3 (0.9)	
Exercise regularly	8310 (84.1)	277 (79.6)	0.025
Height, cm	159.22 ± 7.97	162.08 ± 8.28	< 0.001
Weight, kg	63.07 ± 11.26	65.94 ± 12.17	< 0.001
BMI, kg/m^2^	24.83 ± 3.77	25.01 ± 3.70	0.376
WC, cm	83.37 ± 10.15	84.45 ± 10.61	0.052
SBP, mmHg	142.00 [128.33,160.33]	148.50 [134.42,167.00]	< 0.001
DBP, mmHg	86.54 ± 11.86	88.18 ± 11.39	0.011
FBG, mmol/L	6.14 ± 1.86	6.07 ± 1.36	0.341
HbA1c	5.64 ± 1.09	5.56 ± 0.89	0.150
TC, mmol/L	5.01 [4.37,5.74]	4.97 [4.39,5.66]	0.690
TG, mmol/L	1.28 [0.90,1.87]	1.42 [0.94,2.09]	0.010
LDL-C, mmol/L	2.14 [1.49,2.98]	2.41 [1.67,3.04]	0.011
HDL-C, mmol/L	1.83 [1.41,2.47]	1.55 [1.21,2.13]	< 0.001
Hypertension	5928 (60.0)	238 (68.4)	0.002
Diabetes	1582 (16.0)	51 (14.7)	0.498
Dyslipidemia	2994 (29.3)	127 (36.5)	0.014
Coronary artery disease	377 (3.8)	15 (4.3)	0.636
Cerebrovascular disease	653 (6.6)	30 (8.6)	0.139
Heart rate, bpm	70 [64,78]	67 [60,74]	< 0.001
PR interval, ms	152.30 ± 20.79	222.16 ± 28.88	< 0.001
QRS interval, ms	86 [78,94]	89 [81,98]	< 0.001
QTc interval, ms	424.0 [407.0,443.0]	422.0 [401.0,444.3]	0.075
ECG-LVH	563 (5.7)	26 (7.5)	0.162

Abbreviations: *BMI* body mass index, DBP diastolic blood pressure, *FBG* fasting blood glucose, *HDL-C* high-density lipoprotein cholesterol, *LDL-C* low-density lipoprotein cholesterol, *LVH* left ventricular hypertrophy, *SBP* systolic blood pressure, *TC* total cholesterol, *TG* triglycerides, *WC* waist circumference

Note: Date are presented as mean ± standard deviation, median [upper quartile, lower quartile], or n (%), as appropriate

The overall prevalence of first-degree AVB was 3.4% (348/10231). There was a higher prevalence of first-degree AVB in men than in women (5.2% vs. 2.2%). The highest prevalence was in male participants aged ≥80 years and the lowest in female participants aged 40–49 years. Related details are shown in Table 2.

**Table 2:** Prevalence of first-degree AVB stratified by age and sex among the study participants

Age, years	Male	Female	Total
40–49 (*n* = 1788)	23 (3.8)	14 (1.2)	37 (2.1)
50–59 (*n* = 3100)	52 (4.6)	40 (2.0)	92 (3.0)
60–69 (*n* = 3605)	73 (4.8)	63 (3.0)	136 (3.8)
70–79 (*n* = 1461)	46 (6.9)	15 (1.9)	61 (4.2)
≥80 (*n* = 277)	15 (11.9)	7 (4.6)	22 (7.9)
Total (*n* = 10,231)	209 (5.2)	139 (2.2)	348 (3.4)

Note: Percentages represent the number of subjects with first-degree AVB among the total number of subjects; data are presented as n (%)

Table 3 shows the non-stratified and sex-stratified potential risk factors for first-degree AVB, as identified by the unadjusted regression models. As age increased by 10 years, in the non-stratified analysis, the risk of first-degree AVB increased by 32% (*p* < 0.05). The risk of first-degree AVB in male subjects was significantly higher than in female subjects (OR: 2.37, *ps* < 0.05). In addition, higher height, weight, waist circumference, TG, and LDL-C and hypertension, smoking, and drinking were significantly positively associated with first-degree AVB in the total population (all *ps* < 0.05). In contrast, regular exercise and high HDL-C and heart rate were significantly negatively associated with first-degree AVB (*ps* < 0.05).

**Table 3:** Risk factors for first-degree AVB in unadjusted logistic analyses

	Total		Males		Females	
Variable	OR (95% CI)	*P-*value	OR (95% CI)	*P-*value	OR (95% CI)	*P-*value
Age, per 10 years	1.32 (1.19–1.46)	< 0.001	1.29 (1.12–1.48)	< 0.001	1.26 (1.07–1.49)	0.005
Male vs. female	2.37 (1.90–2.94)	< 0.001	–	–	–	–
Smoking status						
Never smoked	1		1		1	
Former smoker	1.79 (1.28–2.52)	0.001	0.84 (0.55–1.26)	0.835	1.62 (0.59–4.45)	0.354
Current smoker	1.70 (1.34–2.14)	< 0.001	0.80 (0.58–1.10)	0.162	1.56 (0.89–2.73)	0.123
Drinking status						
Never drank	1		1		1	
Moderate drinker	1.73 (1.35–2.22)	< 0.001	1.15 (0.84–1.57)	0.382	1.04 (0.58–1.85)	0.896
Heavy drinker	1.37 (0.97–1.94)	0.076	0.77 (0.52–1.13)	0.185	–	–
Former drinker	0.44 (0.14–1.38)	0.160	0.18 (0.04–0.73)	0.016	1.61 (0.22–11.93)	0.642
Exercise regularly (yes/no)	0.74 (0.57–0.96)	0.026	0.68 (0.48–0.97)	0.031	0.75 (0.50–1.12)	0.159
Height, per 10 cm	1.51 (1.34–1.72)	< 0.001	1.31 (1.07–1.60)	0.009	1.01 (0.78–1.31)	0.942
Weight, per 10 kg	1.22 (1.12–1.33)	< 0.001	1.18 (1.05–1.32)	0.004	1.05 (0.90–1.22)	0.540
BMI, per kg/m^2^	1.01 (0.99–1.04)	0.381	1.04 (1.00–1.08)	0.033	1.01 (0.97–1.06)	0.609
WC, per 10 cm	1.11 (1.00–1.22)	0.045	1.17 (1.03–1.33)	0.020	1.02 (0.87–1.20)	0.808
SBP, per 20 mmHg	1.22 (1.12–1.32)	< 0.001	1.30 (1.16–1.46)	< 0.001	1.16 (1.02–1.32)	0.027
DBP, per 10 mmHg	1.11 (1.02–1.21)	0.013	1.15 (1.02–1.28)	0.019	1.01 (0.88– 1.16)	0.880
FBG, per mmol/L	0.97 (0.91–1.03)	0.353	0.99 (0.92–1.08)	0.874	0.94 (0.84–1.04)	0.239
HbA1c, per 1%	0.93 (0.83–1.04)	0.177	0.95 (0.81–1.11)	0.498	0.98 (0.84–1.14)	0.759
TC, per mmol/L	1.00 (0.91–1.10)	0.983	1.02 (0.90–1.16)	0.776	1.08 (0.95–1.23)	0.241
TG, per mmol/L	1.09 (1.04–1.14)	< 0.001	1.07 (1.00–1.15)	0.037	1.12 (1.05–1.19)	0.001
LDL-C, per mmol/L	1.10 (1.00–1.21)	0.057	1.25 (1.09–1.25)	0.001	1.03 (0.89–1.20)	0.707
HDL-C, per mmol/L	0.65 (0.56–0.75)	< 0.001	0.61 (0.50–0.75)	< 0.001	0.74 (0.60–0.92)	0.006
Hypertension (yes/no)	1.44 (1.15–1.82)	0.002	1.70 (1.25–2.32)	0.001	1.15 (0.81–1.63)	0.438
Diabetes (yes/no)	0.90 (0.67–1.22)	0.498	1.01 (0.68–1.49)	0.978	0.83 (0.52–1.34)	0.452
Dyslipidemia (yes/no)	1.32 (1.06–1.65)	0.014	1.43 (1.07–1.93)	0.017	1.38 (0.98–1.95)	0.063
Coronary artery disease (yes/no)	1.14 (0.67–1.93)	0.636	1.36 (0.70–2.62)	0.361	0.90 (0.37–2.22)	0.823
Cerebrovascular disease (yes/no)	1.33 (0.91–1.96)	0.140	1.55 (1.01–2.38)	0.043	0.51 (0.19–1.39)	0.189
Heart rate, per 10 bpm	0.77 (0.70–0.84)	< 0.001	0.84 (0.74–0.94)	0.004	0.75 (0.64–0.88)	< 0.001
QRS interval, per 10 ms	1.01 (0.99–1.03)	0.223	1.02 (0.98–1.06)	0.321	1.00 (0.97–1.04)	0.840
QTc interval, per 20 ms	0.98 (0.93–1.03)	0.430	1.02 (0.95–1.10)	0.525	1.00 (0.92–1.08)	0.978
ECG-LVH (yes/no)	1.34 (0.89–2.01)	0.164	1.27 (0.74–2.18)	0.393	1.43 (0.77–2.68)	0.260

Abbreviations as in Table 1

Table 4 displays the results of the stepwise multivariate logistic regression analysis, which showed that being male, older, and taller, higher SBP and TG, lower HDL-C and heart rate, and lack of regular exercise were significant independent risk factors for first-degree AVB (all *ps* < 0.05). Further, by performing a sex-stratified analysis, we found that there were different risk factors in males and females. All variables, with the exception of regular exercise, remained significant independent risk factors in males (all *ps* < 0.05). In females, height and SBP were additional variables that were not found to be significant factors (Table 4). We further adjusted for cerebrovascular disease in males, and the results remained unchanged.

**Table 4:** Stepwise multivariate logistic regression analysis of risk factors for first-degree AVB

	Total			Males			Females		
Variable	OR	95% CI	*P*-value	OR	95% CI	*P*-value	OR	95% CI	*P*-value
Age, per 10 years	1.32	1.17–1.49	< 0.001	1.36	1.17–1.59	< 0.001	1.34	1.13–1.59	0.001
Male vs. female	1.72	1.26–2.34	0.001	–	–	–	–	–	–
Exercise regularly (yes/no)	0.73	0.56–0.97	0.030	–	–	–	–	–	–
Height, per 10 cm	1.25	1.06–1.48	0.008	1.37	1.11–1.69	0.003	–	–	–
SBP, per 20 mmHg	1.15	1.05–1.27	0.003	1.23	1.09–1.40	0.001	–	–	–
TG, per 1 mmol/L	1.10	1.05–1.16	< 0.001	1.09	1.02–1.17	0.017	1.13	1.06–1.21	< 0.001
HDL-C, per 1 mmol/L	0.73	0.63–0.84	< 0.001	0.68	0.56–0.84	< 0.001	0.77	0.62–0.95	0.013
Heart rate, per 10 bpm	0.78	0.71–0.86	< 0.001	0.83	0.74–0.94	0.003	0.74	0.63–0.86	< 0.001

Abbreviations as in Table 1

## Discussion

Here, we reported an overall first-degree AVB prevalence of 3.4% (95% CI: 3.0–3.8%) in the Chinese rural population aged ≥40 years. To our knowledge, no epidemiologic studies have reported the prevalence of first-degree AVB in general populations in developing countries. The overall prevalence of first-degree AVB was higher in our study than the published estimates of the overall prevalence of first-degree AVB in general populations in developed countries (1 to 2% ) [4, 5, 10–12].

The variation in the reported prevalences may mainly be due to differences in the subjects’ age range and the cut-off point used to define first-degree AVB. Regarding differences by age, adult participants from Tecumseh, Michigan, USA, [4] aged 40–49, 50–59, 60–69, 70–79, and ≥ 80 years had prevalences of 1.4, 1.2, 4.6, 7.3, and 14.5%, respectively, indicating that the prevalence increases with age, which is similar to our results. Additionally, our stepwise logistic regression results also indicated that age was an independent risk factor (OR: 1.32; *p* < 0.001). Participants in the Framingham Heart Study, [5] the Finnish Social Insurance Institution’s Coronary Heart Disease Study, [10] and the Busselton Heart study [12] were aged > 20 (mean: 47), 30–59 (mean: 44), and 25–84 (mean: 52) years, respectively, with prevalences of 1.6, 2.1, and 1.2%, respectively. However, the present mean age of the participants was 60 years, which may explain the higher prevalence. Further, the prevalence of first-degree AVB is affected by the definition used, and differences in race may also have an important impact [13]. Compared with the aforementioned published estimates (1 to 2%) (among Europeans and Americans with a mean age of about 50 years), the prevalence was similar, at 1.9%, in a Japanese population aged 30–95 years (mean: 50 years) [11]. However, first-degree AVB was defined as a PR interval ≥ 0.20 s in the former studies and ≥ 0.22 s in the Japanese study [11]. Both our population and the Japanese population belong to the Asian race, and our criterion was PR interval > 0.20 s; therefore, it seems reasonable for our AVB prevalence (3.4%) to be higher than those reported in the aforementioned studies. However, when we used a restrictive definition to define first-degree AVB (PR interval ≥ 0.22 s), the prevalence of first-degree AVB was 1.1% (114/10231), which is slightly lower than the prevalence of this ECG pattern in the Japanese population (Additional file 1: Table S1 and Table S2).

Lastly, the prevalence of certain diseases may also affect the prevalence of first-degree AVB. Participants in the Heart and Soul Study with stable coronary artery heart disease were reported to have a prevalence of first-degree AVB of 9.3% (PR internal ≥0.22 s) [23]. In Korean subjects aged > 18 years with hypertension, the prevalence of first-degree AVB was up to 14.3% [14]. In our sample of adults aged > 40 years, the prevalence of hypertension (60.4%) was much higher than that among Koreans aged > 40 years (40.0%) [24]. Thus, it is reasonable that our first-degree AVB prevalence is higher.

We found that the prevalence of first-degree AVB increases with age, which is similar to a finding of a study of a healthy Chinese population that showed that the median PR interval increased with age [25]. In the elderly, electrical and structural remodeling (such as that which occurs in atrial fibrosis) together with calcification and fibrosis of the conduction system, may play an important role in PR interval prolongation [26]. Electrophysiological studies have also confirmed that increased atrial refractoriness and conduction times accompany aging [27, 28]. In addition, in our study, a gender difference in the prevalence of first-degree AVB was found in each age group, with a higher prevalence in males than in females (5.2% vs. 2.2%). The reason is not clear, but the results were consistent with the results of previous studies [4, 5, 10, 14, 23].

Researchers have often reported the characteristics of patients with first-degree AVB in cohort studies [4, 5, 10–12, 14, 23]. However, comprehensive studies on first-degree AVB and associated factors in the general population are limited, especially in China. Here, we compiled demographic and clinical data (including data on BMI, blood pressure, FBG, lipids, etc.) to evaluate the risk factors. Multivariate logistic regression analysis suggested that SBP was an independent risk factor for first-degree AVB. A reasonable explanation is that hypertension may promote elevated intracardiac pressures, causing structural remodeling and changes in atrial electrical function [14, 29]. Strangely, the association between SBP and this ECG pattern was not observed in females. However, the limited number of first-degree AVB patients in the female subgroup (*N* = 139) reduced the statistical power of the analysis. High TG and low HDL-C were also associated with first-degree AVB. This may be because dyslipidemia causes coronary atherosclerosis, which affects the cardiac blood supply, cardiac structure, and electrical conduction. However, as we know, first-degree AVB is associated with coronary artery disease, and dyslipidemia is also recognized as a risk factor for coronary heart disease. Therefore, further studies are needed to confirm whether dyslipidemia is an independent risk factor for first-degree AVB, or whether it is only an independent risk factor for coronary heart disease and therefore associated with first-degree AVB.

Surprisingly, contrary to previous research, which showed that short stature is a risk factor for cardiovascular disease, [30] we found that being tall was a potential risk factor for first-degree AVB. The reason was not clear, but it may be because taller individuals have larger cardiac atria [31]. Additional research is needed to confirm this conclusion. In addition, exercising regularly also showed a significant negative association with first-degree AVB. This may be because of the heightened vagal tone that accompanies physical conditioning (ss in athletes) [32]. Notably, the significant negative association between regular exercise and first-degree AVB was not observed in males or females after sex stratification. The limited number of first-degree AVB patients in the sex-stratified subgroups, especially in the female subgroup, may have affected the results by reducing the statistical power of the analyses.

This study had several limitations. First, the cross-sectional design of the study only allowed assessment of the associations between first-degree AVB and risk factors rather than causal links. Second, although our study included a large number of subjects, these participants were from northeast China and over 40 years old. This may reduce the applicability of our results to other populations. Third, the PR interval includes both the P wave and PR segment. Increased intra-atrial conduction time results in prolongation of the P wave at the expense of the PR segment. Unfortunately, this study did not conduct a detailed analysis on this issue. Lastly, large-sample prospective studies are needed to confirm the results of this study.

## Conclusions

The prevalence of first-degree AVB in rural northeast China is high. The independent risk factors for first-degree AVB included being male, older, and tall, high SBP and TG, and low HDL-C and heart rate. These results provide important insights into first-degree AVB and the predisposing factors, enabling the development of appropriate prevention strategies and design of intensive population-based studies on the prognostic role of first-degree AVB.

## Supplementary information

**Additional file 1: Table S1.** Prevalence of first-degree AVB defined by a restrictive criterion (PR interval ≥ 0.22 s) stratified by age and sex among the study participants. **Table S2.** Stepwise multivariate logistic regression analysis of risk factors for first-degree AVB defined by a restrictive criterion (PR interval ≥ 0.22 s).

## Data Availability

The datasets used and/or analysed during the current study are de-identified and available from the corresponding author on reasonable request.

## Abbreviations

AF: Atrial fibrillation
AVB: Atrioventricular block
BMI: Body mass index
CI: Confidence interval
DBP: Diastolic blood pressure
ECG: Echocardiogram
FBG: Fasting blood glucose
HbA1c: Glycosylated hemoglobin
HDL-C: High-density lipoprotein cholesterol
LDL-C: Low-density lipoprotein cholesterol
OR: Odds ratio
SBP: Systolic blood pressure
TC: Total cholesterol
TG: Triglyceride
