# Supplementary material for: Prevalence of first-degree atrioventricular block and the associated risk factors: a cross-sectional study in rural Northeast China
Source: BMC Cardiovasc Disord. 2019 Oct 7;19:214. doi: 10.1186/s12872-019-1202-4 (PMC6781332; doi:10.1186/s12872-019-1202-4)
Supplement: Supplementary file 1 — Additional file 1: Table S1. Prevalence of first-degree AVB defined by a restrictive criterion (PR interval ≥ 0.22 s) stratified by age and sex among the study participants. Table S2. Stepwise multivariate logistic regression analysis of risk factors for first-degree AVB defined by a restrictive criterion (PR interval ≥ 0.22 s). [file 12872_2019_1202_MOESM1_ESM.docx]

**Table S1.** **Prevalence of first-degree AVB defined by a restrictive criterion (PR interval ≥0.22 s) stratified by age and sex among the study participants.**

| Age, years | Male | Female | Total |
| --- | --- | --- | --- |
| 40–49 (n = 1788) | 7 (1.1) | 5 (0.4) | 12 (0.7) |
| 50–59 (n = 3100) | 13 (1.1) | 14 (0.7) | 27 (0.9) |
| 60–69 (n = 3605) | 26 (1.7) | 19 (0.9) | 45 (1.2) |
| 70–79 (n = 1461) | 18 (2.7) | 5 (0.6) | 23 (1.6) |
| ≥80 (n = 277) | 5 (4.0) | 2 (1.3) | 7 (2.5) |
| Total (n = 10231) | 69 (1.7) | 45 (0.7) | 114 (1.1) |

Note: Percentages represent the number of subjects with first-degree AVB among the total number of subjects; data are presented as n (%).

**Table S2. Stepwise multivariate logistic regression analysis of risk factors for first-degree AVB defined by a restrictive criterion (PR interval ≥0.22 s).**

|  | Total | | |  | Males | | |  | Females | | |
| --- | --- | --- | --- | --- | --- | --- | --- | --- | --- | --- | --- |
| Variable | OR | 95% CI | *P*-value |  | OR | 95% CI | *P*-value |  | OR | 95% CI | *P*-value |
| Age, per 10 years | – | – | – |  | 1.39 | 1.07–1.79 | 0.013 |  | 1.34 | 1.13–1.59 | 0.001 |
| Male vs. female | 2.32 | 1.58–3.40 | <0.001 |  | – | – | – |  | – | – | – |
| Smoking status |  |  |  |  |  |  |  |  |  |  |  |
| Never smoked | – | – | – |  | – | – | – |  | 1 |  |  |
| Former smoker | – | – | – |  | – | – | – |  | 3.86 | 1.17-12.70 | 0.026 |
| Current smoker | – | – | – |  | – | – | – |  | 0.71 | 0.17-2.93 | 0.630 |
| Height, per 10 cm | – | – | – |  | 1.54 | 1.09–2.19 | 0.016 |  | – | – | – |
| SBP, per 20 mmHg | 1.48 | 1.29–1.71 | <0.001 |  | 1.46 | 1.20–1.78 | <0.001 |  | 1.39 | 1.12-1.73 | 0.003 |
| Heart rate, per 10 bpm | 0.82 | 0.70–0.96 | 0.014 |  | 0.80 | 0.65–0.98 | 0.033 |  | – | – | – |

Abbreviations as in table 1.
